# Lattice Boltzmann simulation of alumina-water nanofluid in a square cavity

**DOI:** 10.1186/1556-276X-6-184

**Published:** 2011-02-28

**Authors:** Yurong He, Cong Qi, Yanwei Hu, Bin Qin, Fengchen Li, Yulong Ding

**Affiliations:** 1 School of Energy Science & Engineering, Harbin Institute of Technology, Harbin 150001, China; 2 Institute of Particle Science and Engineering, University of Leeds, Leeds LS2 9JT, UK

## Abstract

A lattice Boltzmann model is developed by coupling the density (D2Q9) and the temperature distribution functions with 9-speed to simulate the convection heat transfer utilizing Al_2_O_3_-water nanofluids in a square cavity. This model is validated by comparing numerical simulation and experimental results over a wide range of Rayleigh numbers. Numerical results show a satisfactory agreement between them. The effects of Rayleigh number and nanoparticle volume fraction on natural convection heat transfer of nanofluid are investigated in this study. Numerical results indicate that the flow and heat transfer characteristics of Al_2_O_3_-water nanofluid in the square cavity are more sensitive to viscosity than to thermal conductivity.

## List of symbols

*c *Reference lattice velocity

*c_s _*Lattice sound velocity

*c_p _*Specific heat capacity (J/kg K)

***e**_α _*Lattice velocity vector

*f_α _*Density distribution function

$f_{\alpha }^{\text{eq}}$ Local equilibrium density distribution function

*F_α _*External force in direction of lattice velocity

***g ***Gravitational acceleration (m/s^2^)

***G ***Effective external force

*k *Thermal conductivity coefficient (Wm/K)

*L *Dimensionless characteristic length of the square cavity

*Ma *Mach number

*Pr *Prandtl number

***r ***Position vector

*Ra *Rayleigh number

*t *Time (s)

*T_α _*Temperature distribution function

$T_{\alpha }^{\text{eq}}$ Local equilibrium temperature distribution function

*T *Dimensionless temperature

*T*_0 _Dimensionless average temperature (*T*_0 _= (*T*_H _+ *T*_C_)/**2**)

*T*_H _Dimensionless hot temperature

*T*_C _Dimensionless cold temperature

***u ***Dimensionless macrovelocity

*u*_c _Dimensionless characteristic velocity of natural convection

*w_α _*Weight coefficient

*x, y *Dimensionless coordinates

## Greek symbols

*β *Thermal expansion coefficient (K^-1^)

*ρ *Density (kg/m^3^)

*ν *Kinematic viscosity coefficient (m^2^/s)

*χ *Thermal diffusion coefficient (m^2^/s)

*μ *Kinematic viscosity (Ns/m^2^)

*φ *Nanoparticle volume fraction

*δ_x _*Lattice step

*δ_t _*Time step *t*

*τ_f _*Dimensionless collision-relaxation time for the flow field

*τ_T _*Dimensionless collision-relaxation time for the temperature field

Δ*T *Dimensionless temperature difference (Δ*T *= *T*_H _- *T*_C_)

Error_1 _Maximal relative error of velocities between two adjacent time layers

Error_2 _Maximal relative error of temperatures between two adjacent time layers

## Subscripts

*α *Lattice velocity direction

avg Average

C Cold

f Fluid

H Hot

nf Nanofluid

p Particle

## Introduction

The most common fluids such as water, oil, and ethylene-glycol mixture have a primary limitation in enhancing the performance of conventional heat transfer due to low thermal conductivities. Nanofluids, using nanoscale particles dispersed in a base fluid, are proposed to overcome this drawback. Nanotechnology has been widely studied in recent years. Wang and Fan [1] reviewed the nanofluid research in the last 10 years. Choi and Eastman [2] are the first author to have proposed the term nanofluids to refer to the fluids with suspended nanoparticles. Yang and Liu [3] prepared a kind of functionalized nanofluid with a method of surface functionalization of silica nanoparticles, and this nanofluid with functionalized nanoparticles have merits including long-term stability and good dispersing. Pinilla et al. [4] used a plasma-gas-condensation-type cluster deposition apparatus to produce nanometer size-selected Cu clusters in a size range of 1-5 nm. With this method, it is possible to produce nanoparticles with a strict control on size by controlling the experimental conditions. Using the covalent interaction between the fatty acid-binding domains of BSA molecule with stearic acid-capped nanoparticles, Bora and Deb [5] proposed a novel bioconjugate of stearic acid-capped maghemite nanoparticle with BSA molecule, which will give a huge boost to the development of non-toxic iron oxide nanoparticles using BSA as a biocompatible passivating agent. Wang et al. [6] showed the method of synthesizing stimuli-responsive magnetic nanoparticles and analyzed the influence of glutathione concentration on its cleavage efficiency. Huang and Wang [7] produced ε-Fe_3_N-magnetic fluid by chemical reaction of iron carbonyl and ammonia gas. Guo et al. [8] investigated the thermal transport properties of the homogeneous and stable magnetic nanofluids containing γ-Fe_2_O_3 _nanoparticles.

Many experiments and common numerical simulation methods have been carried out to investigate the nanofluids. Teng et al. [9] examined the influence of weight fraction, temperature, and particle size on the thermal conductivity ratio of alumina-water nanofluids. Nada et al. [10] investigated the heat transfer enhancement in a horizontal annuli of nanofluid containing various volume fractions of Cu, Ag, Al_2_O_3_, and TiO_2 _nanoparticles. Jou and Tzeng [11] studied the natural convection heat transfer enhancements of nanofluid containing various volume fractions, Grashof numbers, and aspect ratios in a two-dimensional enclosure. Heris et al. [12] investigated experimentally the laminar flow-forced convection heat transfer of Al_2_O_3_-water nanofluid inside a circular tube with a constant wall temperature. Ghasemi and Aminossadati [13] showed the numerical study on natural convection heat transfer of CuO-water nanofluid in an inclined enclosure. Hwang et al. [14] theoretically investigated the natural convection thermal characteristics of Al_2_O_3_-water nanofluid in a rectangular cavity heated from below. Tiwari and Das [15] numerically investigated the behavior of Cu-water nanofluids inside a two-sided lid-driven differentially heated square cavity and analyzed the convective recirculation and flow processes induced by the nanofluid. Putra et al. [16] investigated the natural convection heat transfer characteristics of CuO-water nanofluids inside a horizontal cylinder heated and cooled from both of ends, respectively. Bianco et al. [17] showed the developing laminar forced convection flow of a water-Al_2_O_3 _nanofluid in a circular tube with a constant and uniform heat flux at the wall. Polidori et al. [18] investigated the flow and heat transfer of Al_2_O_3_-water nanofluids under a laminar-free convection condition. It has been found that two factors, thermal conductivity and viscosity, play a key role on the heat transfer behavior. Oztop and Nada [19] investigated the heat transfer and fluid flow characteristic of different types of nanoparticles in a partially heated enclosure. Ho et al. [20] carried out an experimental study to show the natural convection heat transfer of Al_2_O_3_-water nanofluids in square enclosures of different sizes.

The lattice Boltzmann method applied to investigate the nanofluid flow and heat transfer characteristic has been studied in recent years. Hao and Cheng [21] simulated water invasion in an initially gas-filled gas diffusion layer using lattice Boltzmann method to investigate the effect of wettability on water transport dynamics in gas diffusion layer. Xuan and Yao [22] developed a lattice Boltzmann model to simulate flow and energy transport processes inside the nanofluids. Xuan et al. [23] also proposed another lattice Boltzmann model by considering the external and internal forces acting on the suspended nanoparticles as well as mechanical and thermal interactions among the nanoparticles and fluid particles. Arcidiacono and Mantzaras [24] developed a lattice Boltzmann model for simulating finite-rate catalytic surface chemistry. Barrios et al. [25] analyzed natural convective flows in two dimensions using the lattice Boltzmann equation method. Peng et al. [26] proposed a simplified thermal energy distribution model whose numerical results have a good agreement with the original thermal energy distribution model. He et al. [27] proposed a novel lattice Boltzmann thermal model to study thermo-hydrodynamics in incompressible limit by introducing an internal energy density distribution function to simulate the temperature field.

In this study, a lattice Boltzmann model is developed by coupling the density (D2Q9) and the temperature distribution functions with 9-speed to simulate the convection heat transfer utilizing nanofluids in a square cavity.

## Lattice Boltzmann method

In this study, the Al_2_O_3_-water nanofluid of single phase is considered. The macroscopic density and velocity fields are still simulated using the density distribution function.

(1) $$f_{\alpha }\left(r+e_{\alpha }\delta_{t},t+\delta_{t}\right)-f_{\alpha }\left(r,t\right)=-\frac{1}{\tau_{\text{f}}}\left[f_{\alpha }\left(r,t\right)-f_{\alpha }^{\text{eq}}\left(r,t\right)\right]+\delta_{t}F_{\alpha }$$

(2) $$F_{\alpha }=G\cdot \frac{\left(e_{\alpha }-u\right)}{p}f_{\alpha }^{\text{eq}}$$

where τ_f _is the dimensionless collision-relaxation time for the flow field; *e*_α _is the lattice velocity vector; the subscript α represents the lattice velocity direction; *f_α_*(***r**,t*) is the population of the nanofluid with velocity *e*_α _(along the direction α) at lattice ***r ***and time *t*; $f_{\alpha }^{\text{eq}}\left(r,t\right)$ is the local equilibrium distribution function; *δ_t _*is the time step *t*; *F_α _*is the external force term in the direction of lattice velocity; ***G ***= -*β*(*T*_nf_-*T*_0_)***g ***is the effective external force, where ***g ***is the gravity acceleration; *β *is the thermal expansion coefficient; *T *is the temperature of nanofluid; and *T*_0 _is the mean value of the high and low temperatures of the walls.

For the two-dimensional 9-velocity LB model (D2Q9) considered herein, the discrete velocity set for each component *α *is

(3) $$e_{\alpha }=\{\begin{array}{ll} \left(0,0\right) & \alpha =0\\ c\left(\cos\left[\left(\alpha -1\right)\frac{\pi }{2}\right],\sin\left[\left(\alpha -1\right)\frac{\pi }{2}\right]\right) & \alpha =1,2,3,4\\ \sqrt{2}c\left(\cos\left[\left(2\alpha -1\right)\frac{\pi }{4}\right],\sin\left[\left(2\alpha -1\right)\frac{\pi }{4}\right]\right) & \alpha =5,6,7,8 \end{array}$$

where *c *= δ_*x *_/ δ_*t *_is the reference lattice velocity, δ_*x *_is the lattice step, and the order numbers α = 1, ..., 4 and α = 5, ..., 8, respectively, represent the rectangular directions and the diagonal directions of a lattice.

The density equilibrium distribution function is chosen as follows:

(4) $$f_{\alpha }^{\text{eq}}=\rho w_{\alpha }\left[1+\frac{e_{\alpha }\cdot u}{c_{s}^{2}}+\frac{{\left(e_{\alpha }\cdot u\right)}^{2}}{2c_{s}^{4}}-\frac{u^{2}}{2c_{s}^{2}}\right]$$

(5) $$w_{\alpha }=\{\begin{array}{ll} \frac{4}{9} & \alpha =0\\ \frac{1}{9} & \alpha =1,...,4\\ \frac{1}{36} & \alpha =5,...,8 \end{array}$$

where $c_{s}^{2}=\frac{c^{2}}{3}$ is the lattice sound velocity, and *w_alpha _*is the weight coefficient.

The macroscopic temperature field is simulated using the temperature distribution function:

(6) $$T_{\alpha }\left(r+e_{\alpha }\delta_{t},t+\delta_{t}\right)-T_{\alpha }\left(r,t\right)=-\frac{1}{\tau_{\text{T}}}\left[T_{\alpha }\left(r,t\right)-T_{\alpha }^{\text{eq}}\left(r,t\right)\right]$$

where τ_T _is the dimensionless collision-relaxation time for the temperature field.

The temperature equilibrium distribution function is chosen as follows:

(7) $$T_{a}^{\text{eq}}=w_{a}T\left[1+3\frac{e_{a}\times u}{c^{2}}+4.5\frac{{\left(e_{a}\times u\right)}^{2}}{2c^{4}}-1.5\frac{u^{2}}{2c^{2}}\right]$$

The macroscopic temperature, density, and velocity are, respectively, calculated as follows:

(8) $$T=\sum_{\alpha =0}^{8}T_{\alpha }$$

(9) $$\rho =\sum_{\alpha =0}^{8}f_{\alpha }$$

(10) $$u=\frac{1}{\rho }\sum_{\alpha =0}^{8}f_{\alpha }e_{\alpha }$$

The corresponding kinematic viscosity and thermal diffusion coefficients are, respectively, defined as follows:

(11) $$\nu =\frac{1}{3}c^{2}\left(\tau_{\text{f}}-\frac{1}{2}\right)\delta_{t}$$

(12) $$\chi =\frac{1}{3}c^{2}\left(\tau_{\text{T}}-\frac{1}{2}\right)\delta_{t}$$

For natural convection, the important dimensionless parameters are Prandtl number *Pr *and Rayleigh number *Ra *defined by

(13) $$Pr=\frac{\nu }{\chi }$$

(14) $$Ra=\frac{g\beta \Delta TL^{3}Pr}{\nu^{2}}$$

where Δ*T *is the temperature difference between the high temperature wall and the low temperature wall, and *L *is the characteristic length of the square cavity.

Another dimensionless parameter Mach number *Ma *is defined by

(15) $$Ma=\frac{u_{c}}{c_{s}}$$

where $u_{c}=\sqrt{g\beta \Delta TL}$ is the characteristic velocity of natural convection. For natural convection, the Boussinesq approximation is applied; to ensure that the code works in near incompressible regime, the characteristic velocity must be small compared with the fluid speed of sound. In this study, the characteristic velocity is selected as 0.1 times of speed of the sound.

The dimensionless collision-relaxation times τ_f _and τ_T _are, respectively, given as follows:

(16) $$\tau_{\text{f}}=0.5+\frac{MaL\sqrt{3Pr}}{c^{2}\delta t\sqrt{Ra}}$$

(17) $$\tau_{\text{T}}=0.5+\frac{3\nu }{Prc^{2}\delta t}$$

## Lattice Boltzmann model for nanofluid

The fluid in the enclosure is Al_2_O_3_-water nanofluid. Thermo-physical properties of water and Al_2_O_3 _are given in Table 1. The nanofluid is assumed incompressible and no slip occurs between the two media, and it is idealized that the Al_2_O_3_-water nanofluid is a single phase fluid. Hence, the equations of physical parameters of nanofluid are as follows:

**Table 1:** Thermo-physical properties of water and Al_2_O_3 _[29]

Physical properties	Fluid phase (water)	Nanoparticles (Al_2_O_3_)
*ρ *(kg/m^3^)	997.1	3970
*c*_p _(J/kg K)	4179	765
*μ *(m^2^/s)	0.001004	/
*k *(Wm/K)	0.613	25

Density equation:

(18) $$\rho_{\text{nf}}=(1-\phi )\rho_{\text{f}}+\phi \rho_{\text{p}}$$

where *ρ*_nf _is the density of nanofluid, *φ *is the volume fraction of Al_2_O_3 _nanoparticles, *ρ*_bf _is the density of water, and *ρ*_p _is the density of Al_2_O_3 _nanoparticles.

Heat capacity equation:

(19) $$c_{\text{pnf}}=(1-\phi )c_{\text{pf}}+\phi c_{\text{pp}}$$

where *C*_pnf _is the heat capacity of nanofluid, *C*_pf _is the heat capacity of water, and *C*_pp _is the heat capacity of Al_2_O_3 _nanoparticles.

Dynamic viscosity equation [28]:

(20) $$\mu_{\text{nf}}=\frac{\mu_{\text{f}}}{{\left(1-\varphi \right)}^{2.5}}$$

where *μ*_nf _is the viscosity of nanofluid, and *μ*_f _is the viscosity of water.

Thermal conductivity equation [28]:

(21) $$k_{\text{nf}}=k_{\text{f}}\left[\frac{\left(k_{\text{p}}+2k_{\text{f}})-2\varphi (k_{\text{f}}-k_{\text{p}}\right)}{\left(k_{\text{p}}+2k_{\text{f}})+\varphi (k_{\text{f}}-k_{\text{p}}\right)}\right]$$

where *k*_nf _is the thermal conductivity of nanofluid, and *k*_f _is the thermal conductivity of water.

The Nusselt number can be expressed as

(22) $$\text{Nu}=\frac{hH}{k_{\text{nf}}}$$

The heat transfer coefficient is computed from

(23) $$h=\frac{q_{w}}{T_{\text{H}}-T_{\text{L}}}$$

The thermal conductivity of the nanofluid is defined by

(24) $$k_{\text{nf}}=-\frac{q_{w}}{\partial T/\partial x}$$

Substituting Equations (23) and (24) into Equation (22), the local Nusselt number along the left wall can be written as

(25) $$Nu=-\left(\frac{\partial T}{\partial x}\right)\cdot \frac{H}{T_{\text{H}}-T_{\text{L}}}$$

The average Nusselt number is determined from

(26) $${Nu}_{\text{avg}}=\int_{0}^{1}Nu(y)dy$$

## Results and discussion

**Figure 1:**
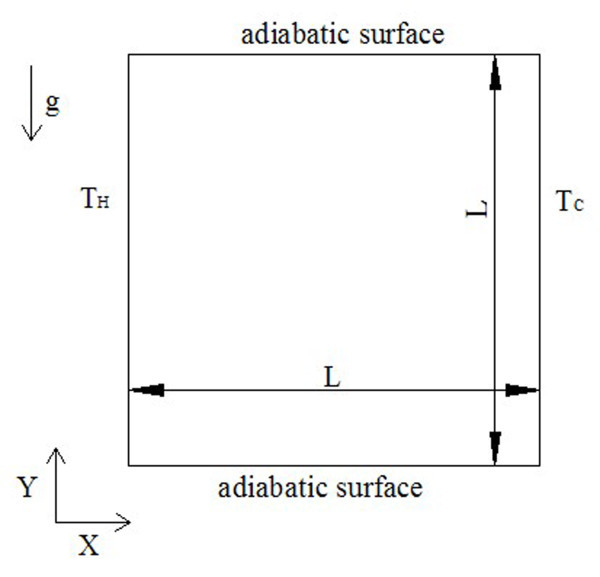
**Schematic of the square cavity**.

The square cavity used in the simulation is shown in Figure 1. In the simulation, all the units are all lattice units. The height and the width of the enclosure are all given by *L*. The left wall is heated and maintained at a constant temperature (*T*_H_) higher than the temperature (*T*_C_) of the right cold wall. The boundary conditions of the top and bottom walls are all adiabatic. The initialization conditions of the four walls are given as follows:

(27) $$\left\{ \begin{array}{l} x=0\;u=0,\;T=1;\;x=1\;u=0,\;T=0\\ y=0\;u=0,\;\partial T/\partial y=0;\;y=1\;u=0,\;\partial T/\partial y=0 \end{array}\right.$$

In the simulation, a non-equilibrium extrapolation scheme is adopted to deal with the boundary, and the standards of the program convergence for flow field and temperature field are respectively given as follows:

(28) $$Error_{1}=\frac{\sqrt{\sum_{i,j}\left\{{\left[u_{x}\left(i,j,t+\delta_{t}\right)-u_{x}\left(i,j,t\right)\right]}^{2}+{\left[u_{y}\left(i,j,t+\delta_{t}\right)-u_{y}\left(i,j,t\right)\right]}^{2}\right\}}}{\sqrt{\sum_{i,j}\left[u_{x}{\left(i,j,t+\delta_{t}\right)}^{2}+u_{y}{\left(i,j,t+\delta_{t}\right)}^{2}\right]}}<\varepsilon_{1}$$

(29) $$Error_{2}=\frac{\sqrt{\sum_{i,j}{\left[T_{x}\left(i,j,t+\delta_{t}\right)-T_{x}\left(i,j,t\right)\right]}^{2}}}{\sqrt{\sum_{i,j}T_{x}{\left(i,j,t+\delta_{t}\right)}^{2}}}<\varepsilon_{2}$$

where *ε *is a small number, for example, for *Ra *= 8 × 10^4^, *ε*_1 _= 10_-7_, and *ε*_2 _= 10_-7_; for *Ra *= 8 × 10^5^, *ε*_1 _= 10_-8_, and *ε*_2 _= 10_-8_.

In the lattice Boltzmann method, the time step *t *= 1.0, the lattice step *δ *= 1.0, the total computational time of the numerical simulation is 100 s, and the data of equilibrium state is chosen in the simulation.

As shown in Table 2, the grid independence test is performed using successively sized grids, 192 × 192, 256 × 256, and 300 × 300 at *Ra *= 8 × 10^5^, *ϕ *= 0.00 (water). From Table 2, it can be seen that the numerical results with grids 256 × 256 and 300 × 300 are more close to those in the literature [20] than with grid 192 × 192, and there is little change in the result as the grid changes from 256 × 256 to 300 × 300. In order to accelerate the numerical simulation, a grid size of 256 × 256 is chosen as the suitable one which can guarantee a grid-independent solution.

**Table 2:** Comparison of the mean Nusselt number with different grids

Physical properties	192 × 192	256 × 256	300 × 300	Literature [20]
*Nu*_avg_	8.367	8.048	7.915	7.704

**Figure 2:**
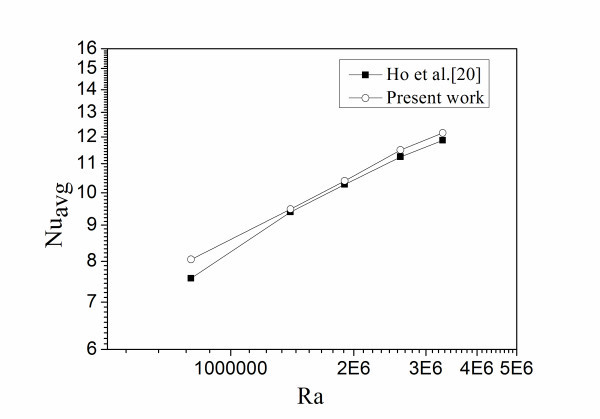
**Comparison of the mean Nusselt number at different Rayleigh numbers**.

To estimate the validity of above proposed lattice Boltzmann model for incompressible fluid, the model is also applied to a nanofluid with nanoparticle volume fraction *ϕ *= 0.00 in a square cavity, and the research object and conditions of numerical simulation are set the same as those proposed in the literature [20]. Figure 2 compares the numerical results with the experimental ones, and a satisfactory agreement is obtained, which indicates that it is feasible to apply the model to incompressible liquids with good accuracy. In Figure 2, there are a few differences because the nanofluid in the simulation is supposed as a single phase, while the real nanofluid is a two-phase fluid. Therefore, the small differences are accepted in the simulation, and the model is appropriate for the simulation of nanofluid.

**Figure 3:**
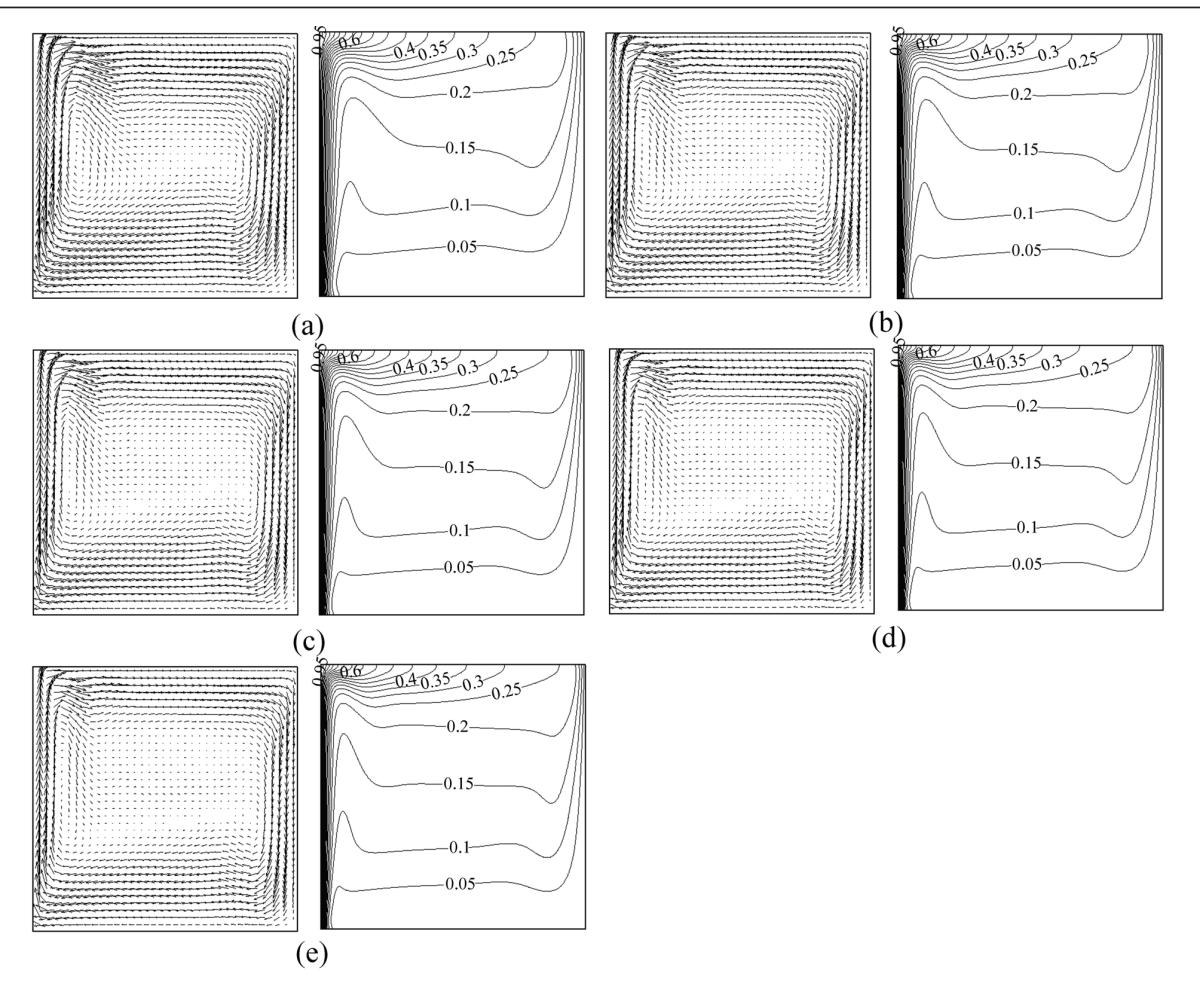
**Velocity vectors (on the left, →0.002) and isotherms (on the right) for Al_2_O_3_-water nanofluid at different Rayleigh numbers**. *φ *= 0.01 **(a) ***Ra *= 8 × 10^5^, **(b) ***Ra *= 1.4 × 10^6^, **(c) ***Ra *= 1.9 × 10^6^, **(d) ***Ra *= 2.6 × 10^6^, **(e) ***Ra *= 3.3 × 10^6^.

Figure 3 illustrates the velocity vectors and isotherms of the Al_2_O_3_-water nanofluid at different Rayleigh numbers with a certain volume fraction of Al_2_O_3 _nanoparticles (*ϕ *= 0.00). It is observed that there are two big vortices in the square cavity at *Ra *= 8 × 10^5^; as the Rayleigh number increases, they are less likely to be observed compared with the condition at smaller Rayleigh numbers. This may be because of the gradually increasing Rayleigh number (corresponding to the increase of the velocity), which causes the nanofluid to rotate mainly around the inside wall of the square cavity. In addition, it can be seen that the temperature isotherms become more and more crooked as *Ra *increases, which illustrates that the heat transfer characteristics transform from conduction to convection.

**Figure 4:**
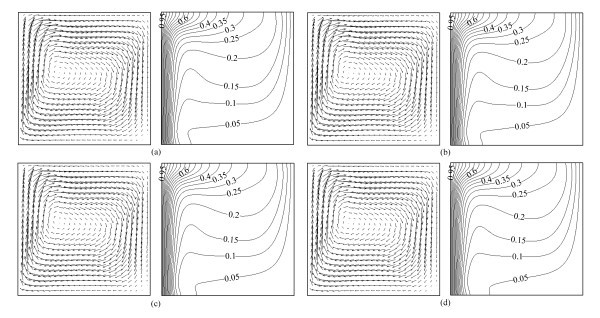
**Velocity vectors (on the left, →0.002) and isotherms (on the right) for Al_2_O_3_-water nanofluid at *Ra *= 8 × 10^4 ^with different volume fractions**. **(a) ***φ *= 0.00, **(b) ***φ *= 0.01, **(c) ***φ *= 0.03, **(d) ***φ *= 0.05.

**Figure 5:**
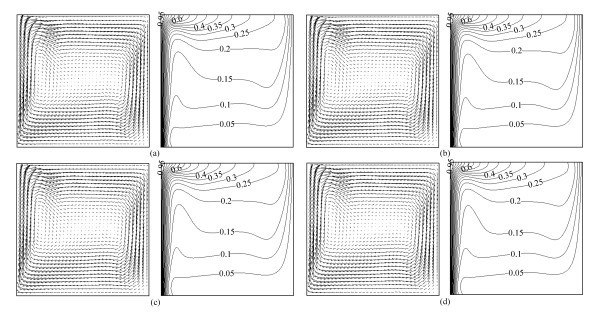
**Velocity vectors (on the left, →0.002) and isotherms (on the right) for Al_2_O_3_-water nanofluid at *Ra *= 8 × 10^5 ^with different volume fractions**. **(a) ***φ *= 0.00, **(b) ***φ *= 0.01, **(c) ***φ *= 0.03, **(d) ***φ *= 0.05.

**Figure 6:**
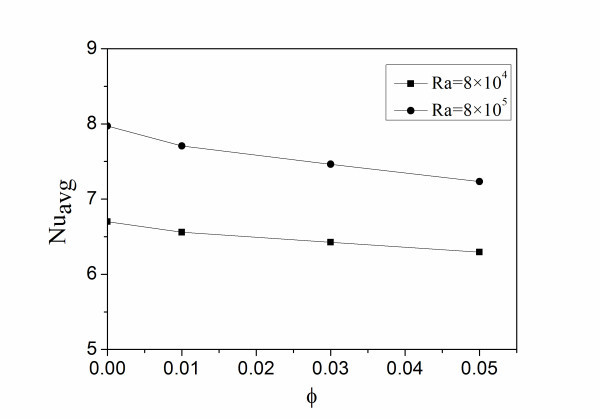
**Average Nusselt numbers at different Rayleigh numbers**.

Figures 4 and 5 present the velocity vectors and isotherms at *Ra *= 8 × 10^4 ^and *Ra *= 8 × 10^5 ^for various volume fractions of Al_2_O_3 _nanoparticles, respectively. There are no obvious differences for velocity vectors and isotherms with different volume fractions of nanoparticles, which is because the volume fractions are so small, it is not significant in this case on comparing with Rayleigh number, and the effect of those volume fractions is negligible. However, it can be seen that there is a little difference on local part of the isotherms, for example, as the volume fraction of Al_2_O_3 _nanoparticles increases, the lowest isotherm in Figure 4 and the second lowest isotherm in Figure 5 become less and less crooked, which indicates that high values of *φ *cause the fluid to become more viscous which causes the velocity to decrease accordingly resulting in a reduced convection. It is more sensitive to the viscosity than to the thermal conductivity for nanofluids heat transfer in a square cavity. This phenomenon can also be observed in Figure 6.

Figure 6 illustrates the relation between the average Nusselt number and the volume fraction of nanoparticles at two different Rayleigh numbers. It is observed that the average Nusselt number decreases with the increase of the volume fraction of nanoparticles for *Ra *= 8 × 10^4 ^and *Ra *= 8 × 10^5^. In addition, it can be seen that the average Nusselt number decreases less at a low Rayleigh number. For the case of *Ra *= 8 × 10^4 ^and *Ra *= 8 × 10^5^, it is indicated that the high values of *φ *cause the fluid to become more viscous which causes reduced convection effect accordingly resulting in a decreasing average Nusselt number, and the flow and heat transfer characteristics of nanofluids are more sensitive to the viscosity than to the thermal conductivity at a high *Ra*.

## Conclusion

A lattice Boltzmann model for single phase fluids is developed by coupling the density and temperature distribution functions. A satisfactory agreement between the numerical results and experimental results is observed.

In addition, the heat transfer and flow characteristics of Al_2_O_3_-water nanofluid in a square cavity are investigated using the lattice Boltzmann model. It is found that the heat transfer characteristics transform from conduction to convection as the Rayleigh number increases, the average Nusselt number is reduced with increasing volume fraction of nanoparticles, especially at a high Rayleigh number. The flow and heat transfer characteristics of Al_2_O_3_-water nanofluid in a square cavity are demonstrated to be more sensitive to viscosity than to thermal conductivity.

## Competing interests

The authors declare that they have no competing interests.

## Authors' contributions

YRH conceived of the study, participated in the design of the program design, checked the grammar of the manuscript and revised it. CQ participated in the design of the program, carried out the numerical simulation of nanofluid, and drafted the manuscript. YWH participated in the design of the program and dealed with the figures. BQ participated in the design of the program. FCL and YLD guided the program design. All authors read and approved the final manuscript.
